# Population pharmacokinetic modeling of ilaprazole in healthy subjects and patients with duodenal ulcer in China

**DOI:** 10.3389/fphar.2023.1306222

**Published:** 2024-01-10

**Authors:** Mengyang Yu, Shupeng Liu, Xiaofei Wu, Hongyun Wang

**Affiliations:** Clinical Pharmacology Research Center, Peking Union Medical College Hospital, State Key Laboratory of Complex Severe and Rare Diseases, NMPA Key Laboratory for Clinical Research and Evaluation of Drug, Beijing Key Laboratory of Clinical PK & PD Investigation for Innovative Drugs, Chinese Academy of Medical Sciences & Peking Union Medical College, Beijing, China

**Keywords:** ilaprazole, population pharmacokinetics, modeling and simulation, proton pump inhibitors, Chinese population

## Abstract

**Aims:** This study aimed to develop a population pharmacokinetic (PopPK) model of ilaprazole in healthy subjects and patients with duodenal ulcer in Chinese and investigate the effect of potential covariates on pharmacokinetic (PK) parameters.

**Methods:** Pharmacokinetic data from 4 phase I clinical trials and 1 phase IIa clinical trial of ilaprazole were included in PopPK analysis. Phoenix NLME 8.3 was used to establish a PopPK model and quantify the effects of covariate, such as demographic data, biochemical indicators and disease state on the PK parameters of ilaprazole. The final model was evaluated by goodness-of-fit plots, bootstrap analysis, and visual predictive check.

**Results:** A two-compartment model with first-order elimination successfully described the pharmacokinetic properties of ilaprazole. In the final PopPK model, body weight and sex were identified as statistically significant covariates for volume of peripheral compartment (V_p_) and clearance of central compartment (CL), respectively, and disease status was also screened as a significant covariate affecting both CL and V_p_. The validation results demonstrated the good predictability of the model, which was accurate and reliable.

**Conclusion:** This is the first population pharmacokinetics study of ilaprazole in the Chinese, and the PopPK model developed in this study is expected to be helpful in providing relevant PK parameters and covariates information for further studies of ilaprazole.

## 1 Introduction

Proton pump inhibitors (PPIs) are the most popular acid suppressive drugs used in clinical practice, and are widely used in the treatment of various acid-related diseases, including peptic ulcer, gastroesophageal reflux disease (GERD), and *Helicobacter pylori* (H. pylori) infection. (Iwakiri et al., 2022; Targownik et al., 2022) Ilaprazole is a novel PPI that discovered by Il-Yang Pharmacy Co. (Seoul, Korea) and developed by Livzon Pharmaceutical Co., Ltd. (Zhuhai, China). It belongs to a class of substituted benzimidazole molecules with the chemical name 2-[[(4-methoxy-3-methyl)-2-pyridinyl] methyl-sulfinyl]-5-(1H-pyrol-1-yl)-1H-benzimidazole (CAS: 172152-36-2) (Kim et al., 2001; Kwon et al., 2001). Ilaprazole exerts gastric acid inhibition by selectively and irreversibly blocking the proton pump (H+/K + -ATPase) in gastric parietal cells. Currently, ilaprazole enteric-coated tablets (indicated for duodenal ulcer and reflux esophagitis in adults) and injection (indicated for peptic ulcer bleeding) have been commercially launched in China.

Notably, the half-life (t_1/2_) of ilaprazole (4.7–5.3 h) is substantially longer than that of first- and second-generation PPIs (0.5–2 h), exhibiting more sustained acid inhibition (Sachs et al., 2006; Shin et al., 2014). Besides, distinguishing from conventional PPIs such as omeprazole, lansoprazole, and pantoprazole (Ben Ghezala et al., 2022), CYP2C19 is not the major metabolizing enzyme catalyzing the formation of metabolites from ilaprazole. Therefore, its pharmacokinetic (PK) characteristics is not affected by CYP2C19 genetic polymorphism, revealing unique clinical advantages (Cho et al., 2012; Seo et al., 2012; Shin et al., 2014). Regarding safety and tolerability, the results of clinical trials indicated that ilaprazole was comparable to omeprazole and rabeprazole in the safety and tolerability profile. The most common drug-related adverse events reported were diarrhea and dysfunction of liver (Wang et al., 2012; Fan et al., 2019). Pharmacokinetics of ilaprazole have been evaluated in many clinical studies and its linear pharmacokinetics was demonstrated, with dose-proportional increases in C_max_ and AUC for both oral administration (10–40 mg) and intravenous infusion administration (5–20 mg) (Shin et al., 2014; Wang et al., 2016a). Nevertheless, there are still significant inter-individual variations in the plasma concentrations of ilaprazole. Differences in PK exposure of ilaprazole between male and female subjects were observed in previous clinical studies with small sample sizes, where AUC and C_max_ were much higher in females than in males (Cho et al., 2012; Cao et al., 2015; Wang et al., 2016a), which can be only partially explained by differences in the demographic. Moreover, It was observed that healthy subjects exhibited higher plasma drug exposure than duodenal ulcers patients (C_max_, 1871.3 vs. 1,540 ng/mL; AUC_0‐∞_, 7.4 vs. 4.9 μg*h/mL), but with comparable efficacy in previous studies (Wang et al., 2019). For proper treatments, it is crucial to comprehend the causes of the disposition variation of ilaprazole *in vivo*.

The population pharmacokinetic (PopPK) model is able to scientifically and effectively characterize the pharmacokinetics of the examined drug and assess the possible factors contributing to pharmacokinetic variability in the population (Goutelle et al., 2022). Currently, PopPK model has been widely used in PPIs such as omeprazole, esomeprazole, and lansoprazole to identify their significant covariates and support rational clinical application or individualized administration (Sakurai et al., 2007; Nagase et al., 2020; Chen et al., 2022). Up to now, there have been no reports on PopPK studies of ilaprazole for the Chinese population, nor on the quantitative analysis of demographic data, biochemical indicators and the influence of disease on drug PK parameters. Previous independent clinical pharmacokinetic studies were limited to the small sample size with non-negligible inter-individual variability. Therefore, it is relevant to review previous studies to quantify the covariance of PK parameters affecting drugs through population pharmacokinetic approaches to facilitate further drug development.

The current study incorporated pharmacokinetic data from several clinical studies in healthy subjects and patients with duodenal ulcers to develop and optimize a population pharmacokinetic model for ilaprazole in Chinese. We aimed to quantify the inter- and intra-individual variability, and clarify the typical covariates that affect the PK properties of ilaprazole, and provide supports for the related future studies.

## 2 Materials and methods

### 2.1 Study populations

Data used to construct the PopPK model in current study were collected from 5 clinical trials of ilaprazole injection, including 4 phase I (No. CTR20132848, No. CTR20140147, No. CTR20150686, No. CTR20150685) clinical trials in healthy subjects, and 1 phase IIa (No. CTR20132846) clinical trial in patients with duodenal ulcer (Wang et al., 2016a; Wang et al., 2016b; Wang et al., 2019). All above studies included in this analysis were reviewed and approved by the Institutional Ethics Committee, registered in the China Clinical Trials Registry Center (chinadrugtrials.org.cn), and conducted in strict adherence to Good Clinical Practice and the Declaration of Helsinki.

The single-dose phase I study (No. CTR20132848) was a randomized, open, four-cycle crossover trial in which 16 healthy subjects were randomized into four groups and were given either oral control drug or intravenous infusion of 5, 10 or 20 mg of ilaprazole during different cycles, after a 1-week washout period, and then separately after crossover. In the multiple-dose phase I study (No. CTR20140147), 10 healthy subjects received a 5-day continuous intravenous infusion of 10 mg ilaprazole once daily. The next studies (No. CTR20150686, No. CTR20150685) were conducted in healthy subjects with a single 30 mg intravenous infusion of ilaprazole, and a 20 mg loading dose combined with a 10 mg maintenance dose for a total of 3 days of intravenous infusion of ilaprazole. In the phase IIa study (No. CTR20132846), 20 patients with duodenal ulcer were randomized to receive a 20 mg loading dose plus a 10 mg maintenance dose of intravenous infusion of ilaprazole once daily or a positive control drug. All the above studies are summarized in Table 1.

**TABLE 1:** Summary of the studies and data used for the Pop PK analysis.

	Study stage/objectives				
	Phase I (CTR20132848)(Single dose study)	Phase I (CTR20140147)(Multiple dosing study)	Phase I (CTR20150686)(High dosing study)	Phase I (CTR20150685)(Loading dose study)	Phase IIa (CTR20132846)(Duodenal ulcer study)
Population	Healthy subjects	Healthy subjects	Healthy subjects	Healthy subjects	Patients with duodenal ulcers^a^
Study design	Single-dose; randomized; open; 4 × 4 crossover	Multiple-dose; randomized; open	Single-dose; randomized; double-blind; parallel; positive controlled	Multiple-dose; randomized; open	Multiple-dose; randomized; open; parallel; positive controlled
Number of subjects	16(16 Ilaprazole^b^)	10 (10 Ilaprazole)	16 (10 Ilaprazole; 6 positive control drug)	12 (12 Ilaprazole)	20 (10 Ilaprazole; 10 positive control drug)
Ilaprazole dose and regimen	5 mg IV infusion, 10 mg IV infusion, or 20 mg IV infusion	10 mg IV infusion, qd	30 mg IV infusion	Day 1: 20 mg IV infusion	Day 1: 20 mg IV infusion
				Day 2, 3: 10 mg IV infusion	Day 2, 3: 10 mg IV infusion
Blood sampling regimen	0 h (predose), 15, 30, 45, 50 min, 1, 1.5, 2, 3, 4, 5, 8, 12, and 24 h post morning dose	day 1: 0 h (predose); 15, 30, 45, 50 min, 1, 1.5, 2, 3, 4, 5, 8, 12, and 24 h	0 h (predose), 15, 30, 45, 50 min, 1, 1.5, 2, 3, 4, 5, 8, 12, and 24 h post morning dose	day 1: 0 h (predose); 15, 30, 45, 50 min, 1, 1.5, 2, 3, 4, 5, 8, 12, and 24 h	0 h (predose), 25, 45, 55 min, 1.5, 2, 3, 5, 8, 12, and 24 h post morning dose on day 1
		day 2: 0 h (predose), 45min			
		day 3: 0 h (predose), 45 min		day 2: 0 h (predose), 45min	
		day 4: 0 h (predose), 45 min			
		day 5: 0 h (predose), 15, 30, 45, 50 min, 1, 1.5, 2, 3, 4, 5, 8, 12, and 24 h		day 3: 0 h (predose), 15, 30, 45, 50 min, 1, 1.5, 2, 3, 4, 5, 8, 12, and 24 h	

Abbreviations: qd: administered once daily. IV: intravenous.

a: ulcer diameter≤15 mm, no combined ulcer bleeding.

b: 1 subjects withdrew after completing the first cycle.

In all of the above studies, the duration of intravenous infusion was 0.75 h.

### 2.2 Sample collection and quantification

Blood samples were collected for clinical pharmacokinetic analysis of ilaprazole in all studies, and the specific sampling designs are summarized in Table 1. To determine the concentration of ilaprazole in human plasma, a validated UPLC-MS/MS method was utilized, with the samples being processed by protein precipitation (Wang et al., 2012). The analytes were chromatographed on Acquity UPLC BEH C18 column (waters, MA, United States), and the detection was performed on Xevo-TQS tandem mass spectrometer coupled with an electro-spray ionization (ESI) source (waters, MA, United States). It is notable that ilaprazole concentration remained linear in the range of 1–1,000 ng/mL, with the lower limit of quantification of 1 ng/mL. After excluding 5 concentrations below the limit of quantification (BLQ) and 7 concentrations not detected (ND), a total of 1,560 plasma concentration from 58 subjects who received intravenous infusion of ilaprazole were included in the final PopPK analysis.

### 2.3 Model development and evaluation

Based on the pooled data from the clinical studies mentioned above, the PopPK model for ilaprazole was constructed using non-linear mixed-effects model of Phoenix NLME (version 8.3; Certara Inc, Princeton, NJ, United States). We developed the PopPK model using the first-order conditional estimation extended least squares (FOCE-ELS) method. The specific modeling steps are as follows.

#### 2.3.1 Development of basic model

For the basic structural model, one-compartment and two-compartment models were tested to fit the data set according to the plasma concentration-time profile of ilaprazole, plotted on a semi-logarithmic scale. Based on the fact that the distribution of the population pharmacokinetic (PK) parameters essentially conforms to a log-normal distribution, the inter-individual variability in pharmacokinetic parameters was estimated by an exponential model (Eq. 1).

$$P_{ik}=P_{poppk}*\exp\;\left(\eta_{\text{ik}}\right)$$ (1)

In Eq. 1, P_ik_ represents the *k*th pharmacokinetic parameter for the *i*th individual, P_popk_ represents the typical value of the parameter in population, and η_ik_ depicts the deviation between the *k*th pharmacokinetic parameter of the *i*th individual and the typical value of the population (i.e., random effect), It is assumed that η follows a normal distribution centered at 0 with variance ω^2^. In addition, intra-individual variation (i.e., residual variability, ε) was tested using additive, multiplicative, and mixed models. Typically, it is assumed that ε are normally distributed with mean 0 and variance σ^2^.

The algorithm used for this model is first-order conditional estimation-extended least squares (FOCE-ELS). The final basic model was determined by comparing visual inspection diagnostic plots, objective function values (OFV), Akaike information criterion (AIC), and Bayesian information criterion (BIC), where smaller AIC and BIC values indicate that the model better balances the goodness of fit and parameter complexity, while lower OFV values indicate better model fit results.

#### 2.3.2 Development of covariate model

After completing the construction of basic model, covariates were screened by stepwise covariate model. One part of the covariates used for model analysis was obtained directly from electronic medical records, including sex, disease status (healthy or duodenal ulcers), age, height (HT), weight (WT), total protein (TP), albumin (ALB), platelet count (PLT), alanine aminotransferase (ALT), aspartate aminotransferase (AST), total bilirubin (TBIL), and creatinine (Cr), while the other part was further calculated using formulas, such as body mass index (BMI) and creatinine clearance (CLCr). BMI was calculated by a formula (Eq. 2) approved by the World Health Organization (WHO) and CLCr was calculated by the Cockcroft-Gault formula (Eq. 3 and Eq. 4) (Keys et al., 1972; Cockcroft and Gault, 1976).

$$BMI=\frac{Weight\left(kg\right)}{{\;Height\left(m\right)}^{2}}$$ (2)

$$For\;Male:CLCr\left(mL/\min\right)=\frac{\left(140-Age\left(year\right)\right)*Weight\left(kg\right)}{72*Serum\;Creatinine\left(mg/dL\right)}$$ (3)

$$For\;Female:CLCr\left(mL/\min\right)=\frac{\left(140-Age\left(year\right)\right)*Weight\left(kg\right)}{72*Serum\;Creatinine\left(mg/dL\right)}*0.85\;$$ (4)

The effect of continuous covariates and categorical covariates for the *k*th pharmacokinetic parameter of the *i*th individual were described by Eq. 5 and Eq. 6, respectively.

$$P_{ik}=P_{poppk}*\exp\;\left(\eta_{\text{ik}}\right)*{\left({COVR}_{i}/{COVR}_{median}\right)}^{\theta_{COVR}}$$ (5)

$$P_{ik}=P_{poppk}*\exp\left(\eta_{\text{ik}}\right)*\exp\left(\theta_{COVR}*{COVR}_{i}\right)$$ (6)

In Eq. 5, 6, COVR_median_ is the population median value of covariate, COVR_i_ is the covariate value of the *i*th individual, and θ_COVR_ is a fixed-effect factor.

However, not all of the above covariates were used to construct the final covariate model. A collinearity analysis of the covariates was conducted. In cases where two covariates had significant impacts on the same PK parameter and exhibited a strong correlation (*R* ^2^ > 0.5), only one of them was retained in the model. After that, the stepwise method, including forward inclusion and backward elimination, was used to systematically screen covariates that may affect PK parameters. During the forward inclusion process, covariates were added in the model if the decrease in OFV exceeded 6.635 (*p* < 0.01). Subsequently, during the backward elimination process, covariates were retained in the model if the increase in OFV was less than 10.828 (*p* < 0.001), otherwise they were excluded. In addition, the correlation between PK parameters should be clarified to determine whether a covariance model needs to be constructed.

#### 2.3.3 Validation of the final model

The validity of final model was confirmed by goodness-of-fit plots (GOF), visual predictive check (VPC), and non-parametric bootstrap. GOF diagnostic plots consist of the following: dependent variable *versus* population prediction plot (DV-PRED), dependent variable *versus* individual prediction plot (DV-IPRED), conditional weighted residuals errors *versus* time after dose plot (CWRES-TAD), and conditional weighted residuals errors *versus* population prediction plot (CWRES-PRED). The fit of model was evaluated by observing the distribution trend, range and homogeneity of these GOF plots. Bootstrap is a resampling technique used to evaluate the accuracy of parameter estimation and the robustness of the model. During the analysis process, samples were repeatedly sampled using a random sampling technique with replacement, the generated dataset was also modeled. The median values and 95% confidence intervals of the obtained pharmacokinetic parameters were compared with the typical values of the parameters in final model. VPC generates a virtual dataset through Monte Carlo simulation. The predictive performance of the model was evaluated by comparing the observations and predictions at the fifth, 50th, and 95th percentiles.

## 3 Results

### 3.1 Baseline of demographics and clinical characteristics

The current study included data from 58 subjects (48 healthy subjects and 10 duodenal ulcer patients) in 5 clinical trials of ilaprazole, with a total of 1,560 valid plasma concentration points, 48.3% of the subjects were female. In the clinical trial conducted among duodenal ulcer patients, the exclusion criteria included ‘history of alcohol abuse, drug abuse, or other factors affecting drug metabolism’, therefore, all the duodenal ulcer patients participating in the study had no history of smoking and drinking. The PopPK model for ilaprazole was successfully constructed, and the baseline of demographic and clinical characteristics of subjects were summarized in Table 2.

**TABLE 2:** Baseline of demographics and clinical characteristics.

Baseline characteristics	Study stage/objectives					
	Phase I (single dose study)N = 16	Phase I (multiple dosing study)N = 10	Phase I (high dosing study)N = 10	Phase I (loading dose study)N = 12	Phase IIa (duodenal ulcer study)N = 10	TotalN = 58
Demographic data						
Age, year (median, IQR)	24 (22.3, 27.3)	25 (22, 31.3)	24.5 (22, 27.5)	25.5 (23, 28)	46 (38.3, 50.5)	25 (23, 31)
Gender (female) (n, %)	8 (50)	5 (50)	5 (50)	6 (50)	4 (40)	28 (48.3)
HT, cm (median, IQR)	170 (161, 174.8)	169 (163, 177.3)	166 (159.7, 171.9)	166.3 (161.6, 170.2)	168.5 (159.8, 170)	168 (160.5, 172.3)
WT, kg (median, IQR)	62.5 (54.3, 69.8)	62 (55.5, 67)	58.1 (53.1, 62.6)	59.5 (57.2, 65.4)	63 (58.8, 65)	60.6 (55.8, 65.1)
BMI, kg/m^2^ (median, IQR)	21.6 (20.8, 22.8)	21.2 (20.9, 22.7)	21.3 (19.3, 22.6)	22.2 (20.3, 23.9)	22.3 (21.2, 23.6)	21.6 (20.8, 22.7)
Clinical data						
TP, g/L (median, IQR)	75 (71.7, 77.8)	72.8 (69.5, 74.6)	72.5 (70.3, 73.5)	73.4 (70.9, 78)	71.2 (70.5, 73.2)	72.8 (70.7, 75.7)
ALB, g/L (median, IQR)	46.9 (45, 49.4)	45.5 (43.1, 48.1)	44.7 (42.6, 46.2)	45.7 (43.3, 48.2)	45.3 (43.2, 47.6)	45.4 (43.6, 47.5)
PLT, 10^9^/L (median, IQR)	217.5 (191.3, 264)	231 (204.3, 293.3)	221 (187.3, 268.3)	218.5 (188.5, 270.3)	222 (185.5, 254.3)	220 (196, 262.3)
ALT, U/L (median, IQR)	13.8 (9.8, 17.7)	13.9 (8.8, 18.9)	10.8 (9.0, 20.0)	13.3 (11.6, 17.6)	19.6 (15.6, 27.0)	14.1 (9.8, 19.2)
AST, U/L (median, IQR)	16.9 (15.0, 20.5)	16.2 (15.5, 23.0)	16.8 (14.0, 20.6)	18.3 (14.7, 21.1)	22.6 (17.6, 25.7)	17.9 (15.2, 21.6)
TBIL, umol/L (median, IQR)	11.9 (8.5, 13.7)	12.1 (8.2, 14.7)	8 (7.2, 11.9)	10.8 (8.1, 13.8)	12.4 (9.1, 16.2)	11.6 (8.2, 13.6)
Cr, umol/L (median, IQR)	66.5 (64, 81.8)	77.7 (58.5, 85)	71.2 (55.1, 77.1)	65.6 (49.8, 74)	72.5 (57.1, 82.7)	67.4 (58.5, 79.6)
CLCr, mL/min (median, IQR)	114.9 (94.8, 127.8)	118.4 (101.7, 121.4)	118.5 (107.1, 120.8)	126 (118.0, 142.4)	94.8 (88.4, 115)	118.1(98.2, 124.1)

IQR, interquartile range; HT, height weight (WT); WT, weight; BMI, body mass index; TP, total protein; ALB, albumin; PLT, platelet count; ALT, alanine aminotransferase; AST, aspartate aminotransferase; TBIL, total bilirubin; Cr, creatinine; CLCr, creatinine clearance.

### 3.2 Population pharmacokinetic model

In this study, we evaluated the applicability of one-compartment model and two compartment model for fitting the plasma concentrations of ilaprazole according to the objective function values (OFV) and GOF (Supplementary Figure S1, S2). Observed ilaprazole plasma concentrations were best described by a two-compartment model with first-order elimination (ΔOFV = 929.103 compared to one-compartment model), with parameters including volume of central compartment (V), clearance of central compartment (CL), volume of peripheral compartment (V_p_), and clearance of peripheral compartment (CL_p_). An exponential model was used to describe inter-individual variability and a multiplicative residual error model was used to characterize intra-individual variability. All parameters were estimated with high precision with RSE<20% (Table 3), and CL_p_ was fixed due to its high η-shrinkage (84%).

**TABLE 3:** Population pharmacokinetic parameter estimates for final model of ilaprazole and bootstrap results (n = 1,000).

Parameter	Final model			Bootstrapping		
	Estimate	RSE%	95% CI	Median	RSE%	95% CI
Typical value parameter of population						
V (L)	6.795	5.230	6.098–7.492	6.819	5.784	6.183–7.633
V_p_ (L)	5.544	7.182	4.763–6.326	5.480	8.420	4.625–6.350
CL (L/h)	3.394	3.225	3.180–3.609	3.394	3.300	3.179–3.621
CL_p_ (L/h)	13.086	16.896	8.749–17.423	12.946	18.070	8.239–17.389
Covariable effect						
Sex effect on CL (Sex = 1)	−0.213	−23.398	−0.311–0.115	−0.214	−24.208	−0.313–0.111
WT effect on V_p_	1.545	13.714	1.129–1.960	1.573	13.558	1.168–2.030
Disease status effect on CL (Disease status = 1)	0.290	23.751	0.155–0.425	0.288	25.352	0.147–0.441
Disease status effect on V_p_ (Disease status = 1)	0.356	14.707	0.253–0.459	0.356	16.472	0.241–0.481
inter-individual variability						
ω^2^_V_ (shrinkage)	0.013 (34.0%)	31.284	0.005–0.021	0.013	36.269	0.004–0.022
ω^2^_Vp_ (shrinkage)	0.032 (1.82%)	21.763	0.018–0.046	0.033	23.588	0.018–0.048
ω^2^_CL_ (shrinkage)	0.059 (24.4%)	25.051	0.030–0.089	0.057	25.181	0.029–0.085
ω^2^_CLp_	Fixed					
Residual variability						
σ_mult_	0.184	9.131	0.151–0.217	0.182	8.903	0.149–0.213

RSE%, relative standard error; CI, confidence interval; V, volume of central compartment; CL, clearance of central compartment; V_P_, volume of peripheral compartment; CL_P_, clearance of peripheral compartment; Sex = 1 for female; Disease status = 1 for duodenal ulcer.

In the covariate analysis, two covariates with a correlation coefficient greater than 0.5 were refrained from containing simultaneously in order to avoid the covariate collinearity. The distribution and correlation of continuous covariates were shown in Supplementary Figure S3. Among them, the correlation coefficients between WT and HT, WT and BMI, ALB and TP, and AST and ALT were 0.810, 0.638, 0.636, and 0.767, respectively. Therefore, only WT, TP, AST, and the rest of the variables that were not correlated with each other were retained for covariate screening. Eventually, disease status, weight and sex were included in the final model. The Disease status effect on CL and V_p_ were 0.290 (RSE 23.751%) and 0.356 (RSE 14.707%) respectively. The sex effect on CL and the weight effect on V_p_ were −0.231 (RSE −23.398%) and 1.545 (RSE 13.714%), respectively. The results of the stepwise procedures including forward inclusion and backward elimination were presented in Table 4. The parameter estimates for the final PopPK model were presented in Table 3, and the final model were described as follows (Eq 7, 8, 9):

$$V=V*\exp\left(\eta_{V}\right)$$ (7)

$$CL=CL*\exp\left(-0.213*Sex\right)*\exp\left(0.29*Disease\;status\right)*\exp\left(\eta_{CL}\right)$$ (8)

$$V_{p}=V_{p}*{\left(\frac{Weight}{60.6}\right)}^{1.545}*\exp\left(0.356*Disease\;status\right)*\exp\left(\eta_{V_{p}}\right)$$ (9)

**TABLE 4:** Results of the forward and backward stepwise procedure.

Step	Covariate screening	OFV	∆OFV	Comments
1	None	16368.268	—	Base model
Forward inclusion Add covariates that result in the decrease of ∆OFV>6.635 (*p* < 0.01)				
2	V_p_-Weight	16334.847	33.421	
3	V_p_-Weight/V_p_-Disease status	16317.904	16.943	
4	V_p_-Weight/V_p_- Disease status/CL-Sex	16303.165	14.739	
5	V_p_-Weight/V_p_-Disease status/CL-Sex/CL- Disease status	16292.018	11.147	
6	Vp-Weight/Vp-Disease status/CL-Sex/CL- Disease status/V-TBIL	16281.920	10.098	Full model
Backward elimination Subtract covariates that result in the increase of ∆OFV>10.828 (*p* < 0.001)				
7	Vp-Weight/Vp-Disease status/CL-Sex/CL-Disease status	16292.018	10.098	Final model

OFV, objective function value; ∆OFV, variation of objective function value.

Where 6.795L and 5.544 L were the typical value of V and V_p_, 3.394 L/h and 13.086 L/h were the typical value of CL and CL_p_. 1.545 referred to the effect of body weight on V_p_, the median weight was 60.6 kg −0.213 referred to the effect of sex on CL, sex = 1 for females. 0.29 and 0.356 referred to the effect of disease status on CL and V_p_, respectively. Disease status = 1 for duodenal ulcer.

### 3.3 Model validation and simulation

**FIGURE 1:**
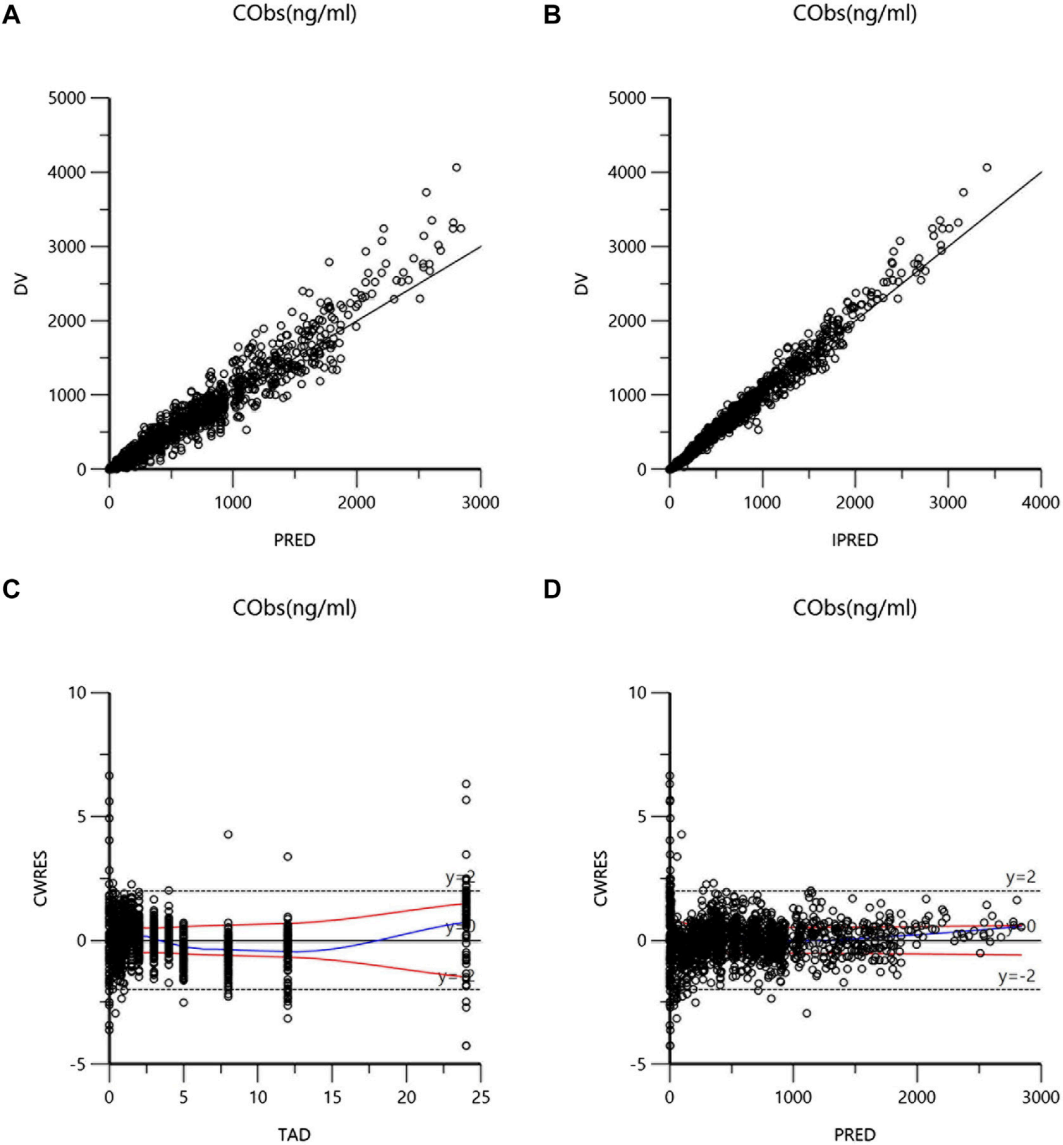
Goodness-of-fits plots for the final model: **(A)** dependent variable *versus* population prediction plot (DV-PRED) of ilaprazole; **(B)** dependent variable *versus* individual prediction plot (DV-IPRED) of ilaprazole; **(C)** conditional weighted residuals errors *versus* time after last dose plot (CWRES-TAD) of ilaprazole; **(D)** conditional weighted residuals errors *versus* population prediction plot (CWRES-PRED) of ilaprazole.

**FIGURE 2:**
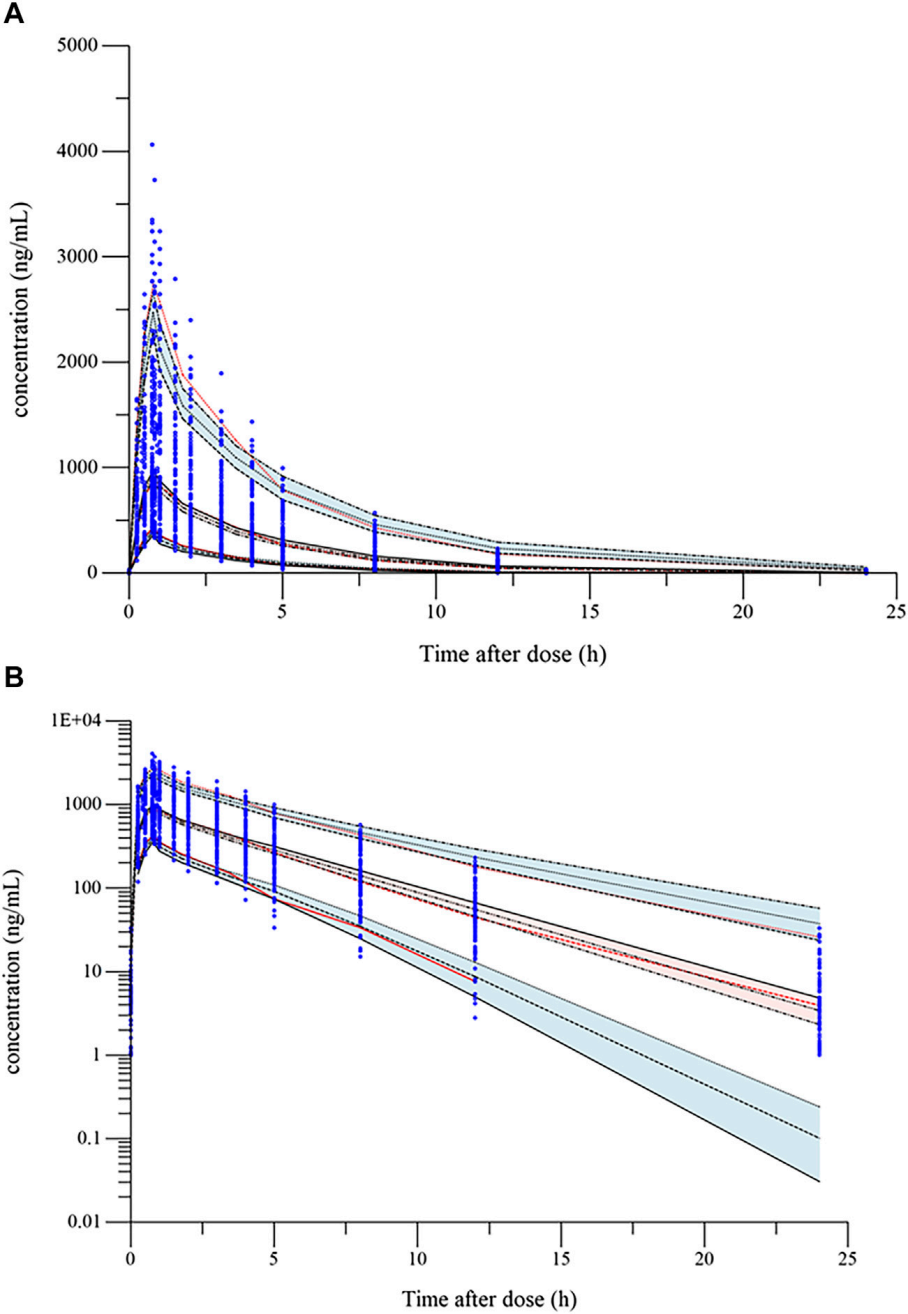
Visual predictive check (VPC) plot of the final model: **(A)** VPC plot (constant plot); **(B)** VPC plot (logarithmic plot). The blue dots are the observed concentrations. The lower, middle, and upper red dotted lines represent the fifth, 50th, and 95th percentiles of the observed data, respectively. The upper, middle, and lower black dotted lines represent the 95th, 50th, and fifth percentiles of the simulated concentrations, respectively; red and blue shaded areas are their model-predicted 90% CIs.

The goodness-of-fit plots of the final model were presented in Figure 1. The data points in DV-PRED and DV-IPRED diagnostic plots were uniformly distributed on both sides of the reference line (y = x), indicating that the individual and population predicted values of the model had a good correlation with the observed values, which suggests that the model can fit the observed values well. In the CWRES-PRED and CWRES-TAD diagnostic plots, most CWRES values were randomly distributed between +2 and −2 with no obvious bias, indicating good model efficacy. Visual predictive check (VPC) plot was used to evaluate the predictive ability of the final model, as shown in Figure 2. The 90% confidence intervals of the 5, 50, and 95% quartiles of the predicted data generated by the simulation had a similar trend with the corresponding quartiles of the clinical observations. The vast majority of DVs were contained within the confidence intervals simulated by the model, indicating that the final model was well predictive and well characterized the pharmacokinetics of ilaprazole. In addition, as shown in Table 3, the estimates of all pharmacokinetic parameters of the final model were contained in the 95% confidence intervals calculated by the Bootstrap method. All the above results showed that the final model had good prediction performance, which was accurate and reliable.

**FIGURE 3:**
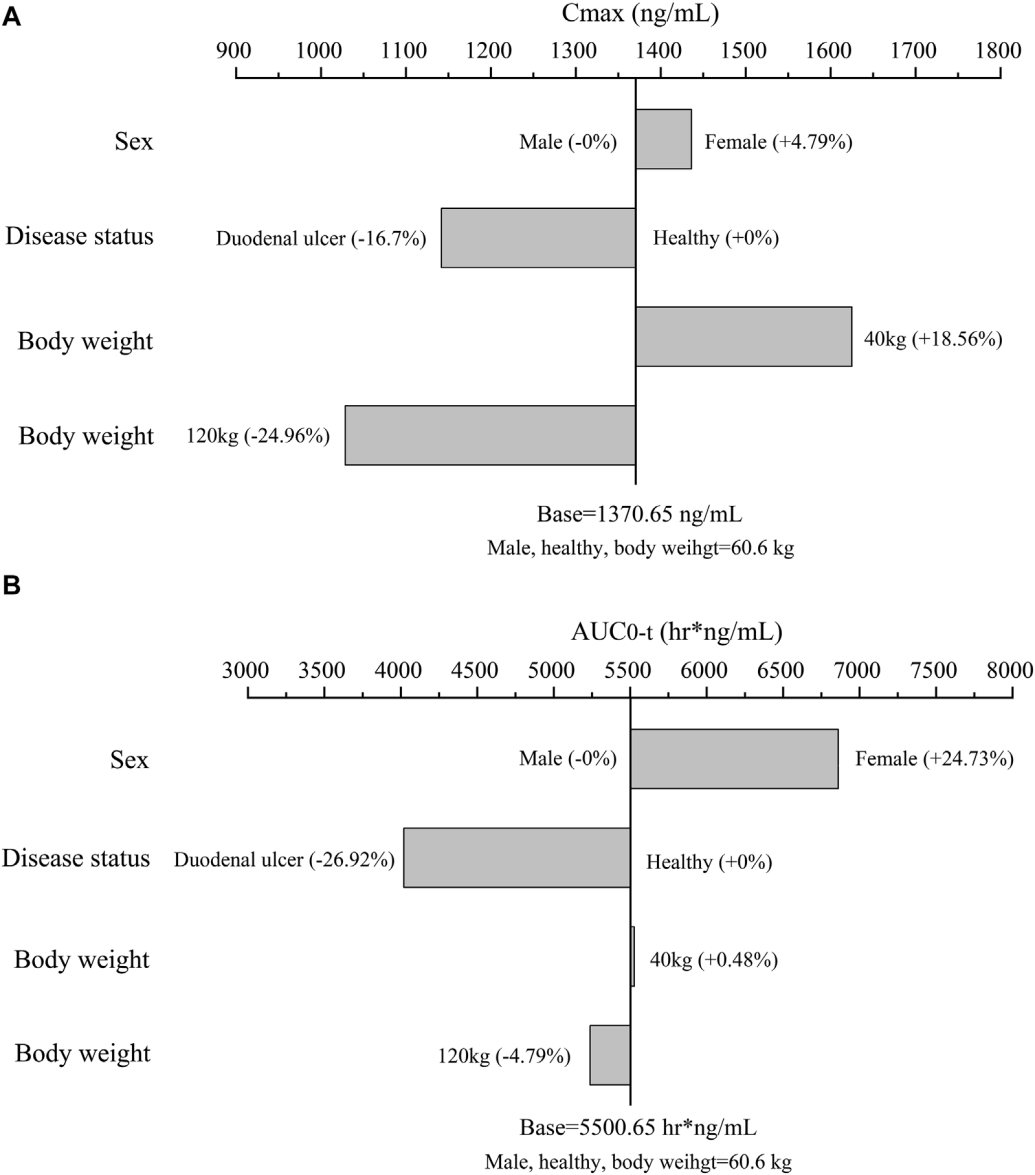
The effect of significant covariates on ilaprazole’s exposure: **(A)** C_max_; **(B)** AUC_0-t_; the typical subject is a healthy male volunteer with body weight of 60.6 kg (Median body weight of the study population). For body weight (a continuous covariate), overweight (120 kg) and underweight (40 kg) were assessed. For sex and disease status (categorical variables), we assessed them according to category.

Using the final model, simulations of single intravenous infusion of ilaprazole 20 mg were performed. The effect of significant covariates on ilaprazole’s exposure (C_max_ and AUC_0-t_) was presented in Figure 3. The results showed that C_max_ and AUC_0-t_ were 4.79% and 24.73% higher in females than in males after ilaprazole treatment, respectively. In contrast to healthy individuals, patients with duodenal ulcers showed a 16.7% decrease in C_max_ and a 26.92% decrease in AUC_0-t_. Furthermore, body weight had a mild impact on AUC_0-t_ (∼5%) and affected C_max_ by less than 25%.

## 4 Disscussion

As proton pump inhibitor drug, studies focusing on the mechanism of action of ilaprazole have been extensive and in-depth. Non-etheless, studies exploring the inter-individual variability of ilaprazole in the population are still limited. Hence, the current study firstly developed and optimized a PopPK model for ilaprazole in Chinese, assessing the effect of demographic data, biochemical indices, and disease status on its PK parameters. The final model successfully characterized the PK properties of ilaprazole and quantified the effect of covariates on its disposition. Body weight and sex were included in the final model as significant covariates, where the former affected volume of peripheral compartment (V_p_) and the latter affected clearance of central compartment (CL). Disease status was also screened as a significant covariate to be added in the final model, affecting both CL and V_p_.

Ilaprazole is more lipophilic (LogP = 3.04) compared to other PPIs, with high plasma protein binding capacity of more than 97%. Biodistribution study after intravenous injection of ^14^C-ilaprazole in rats demonstrated that it distributed in most tissues/organs and the radioactive signal of ilaprazole can still be observed in the stomach after 24 h (Shen et al., 2020; Jiang et al., 2021). In this study, analysis of covariates indicated that the peripheral volume of distribution of ilaprazole increased with increasing body weight, which is consistent with the finding in previous studies that the volume of distribution of lipophilic drugs tends to correlate with total body weight (Hanley et al., 2010; Morrish et al., 2011). Additionally, Sex was identified as a covariate affecting the clearance of ilaprazole, with lower clearance in females. Higher exposures and lower clearance rates in women than in men have been observed in several previous clinical trials (Wang et al., 2016a; Wang et al., 2016b; Wang et al., 2019). Mechanically, the major metabolite of ilaprazole (ilaprazole sulfone) was predominantly catalyzed by CYP3A4/5. The CYP isoenzymes seem to be more active in men than in women, as are conjugation reactions, which supported the findings of this study (Gleiter and Gundert-Remy, 1996; Tanaka, 1999). In addition, data from 10 patients with duodenal ulcers were included in this PopPK analysis, and covariate screening demonstrated that disease status (duodenal ulcer) had effect on both of V_P_ and CL of ilaprazole. Patients with duodenal ulcers tended to have a wider peripheral distribution volume and higher clearance. Whether the above effects have an impact on drug efficiency needs to be further analyzed in conjunction with the pharmacodynamic (PD) model.

Inevitably, there are some limitations of this analysis. The amount of data used for model construction was limited and only one clinical study conducted in patients was included. This is due to the fact that pharmacokinetic plasma samples of ilaprazole injection were only collected in phase I and IIa, all currently available PK data were included in the PopPK model. As clinical studies on ilaprazole continue, it is expected that more data from subsequent studies can be included and refreshed the current model to provide recommendations and guidance for further clinical applications. Regarding the under-prediction observed in the visual predictive check (VPC) plot at high concentration data points, this was primarily noted in the 30 mg (high-dose) group. This group, included in the clinical study data of only 10 subjects, represents a small fraction of the total dataset, which may contribute to the model’s suboptimal fitting for this subset. Notably, the standard doses specified in the ilaprazole injection package insert are 20 mg (initial dose) and 10 mg (maintenance dose). Our model demonstrates good fitting within the dosage range in actual clinical practice. Therefore, the model still retains considerable practical utility despite the noted limitations.

## 5 Conlusion

In conclusion, the current study firstly established a population pharmacokinetics model of ilaprazole. A two-compartment disposition model with first-order elimination accurately describes the plasma concentrations of ilaprazole. The results indicated that body weight and sex were significant covariates affecting drug peripheral distribution volume and clearance, respectively, and disease status was also a significant covariate affecting both of the above parameters. The PopPK model developed in this study is expected to be helpful in providing relevant PK parameters and covariates information for further studies of ilaprazole.

## Data Availability

The original contributions presented in the study are included in the article/Supplementary Materials, further inquiries can be directed to the corresponding author.

## References

[B1] Ben Ghezala I. Luu M. Bardou M. (2022). An update on drug-drug interactions associated with proton pump inhibitors. Expert Opin. drug metabolism Toxicol. 18 (5), 337–346. 10.1080/17425255.2022.2098107 35787720

[B2] Cao S. Zhou G. Chen Y. Guo D. Tan Z. Fan L. (2015). Gender, but not CYP2C19 genotypes and CYP3A phenotypes, is a major determinant of ilaprazole pharmacokinetic. Am. J. Life Sci. 3 (1-4), 14–20. 10.11648/J.AJLS.S.2015030104.13

[B3] Chen K. Luo P. Yang G. Zhu S. Deng C. Ding J. (2022). Population pharmacokinetics of omeprazole in obese and normal-weight adults. Expert Rev. Clin. Pharmacol. 15 (4), 461–471. 10.1080/17512433.2022.2075343 35522794

[B4] Cho H. Choi M. K. Cho D. Y. Yeo C. W. Jeong H. E. Shon J. H. (2012). Effect of CYP2C19 genetic polymorphism on pharmacokinetics and pharmacodynamics of a new proton pump inhibitor, ilaprazole. J. Clin. Pharmacol. 52 (7), 976–984. 10.1177/0091270011408611 21593280

[B5] Cockcroft D. W. Gault M. H. (1976). Prediction of creatinine clearance from serum creatinine. Nephron 16 (1), 31–41. 10.1159/000180580 1244564

[B6] Fan L. Xianghong Q. Ling W. Ying H. Jielai X. Haitang H. (2019). Ilaprazole compared with rabeprazole in the treatment of duodenal ulcer: a randomized, double-blind, active-controlled, multicenter study. J. Clin. Gastroenterol. 53 (9), 641–647. 10.1097/MCG.0000000000001186 30789856

[B7] Gleiter C. H. Gundert-Remy U. (1996). Gender differences in pharmacokinetics. Eur. J. drug metabolism Pharmacokinet. 21 (2), 123–128. 10.1007/BF03190260 8839685

[B8] Goutelle S. Woillard J. B. Neely M. Yamada W. Bourguignon L. (2022). Nonparametric methods in population pharmacokinetics. J. Clin. Pharmacol. 62 (2), 142–157. 10.1002/jcph.1650 33103785

[B9] Hanley M. J. Abernethy D. R. Greenblatt D. J. (2010). Effect of obesity on the pharmacokinetics of drugs in humans. Clin. Pharmacokinet. 49 (2), 71–87. 10.2165/11318100-000000000-00000 20067334

[B10] Iwakiri K. Fujiwara Y. Manabe N. Ihara E. Kuribayashi S. Akiyama J. (2022). Evidence-based clinical practice guidelines for gastroesophageal reflux disease 2021. J. gastroenterology 57 (4), 267–285. 10.1007/s00535-022-01861-z PMC8938399 35226174

[B11] Jiang X. Shen T. Jin Z. Li Q. Qiu W. Li C. (2021). An analysis of the biopharmaceutical behaviour of proton pump inhibitors with different physicochemical properties. Life Sci. 286, 120042. 10.1016/j.lfs.2021.120042 34678262

[B12] Keys A. Fidanza F. Karvonen M. J. Kimura N. Taylor H. L. (1972). Indices of relative weight and obesity. J. chronic Dis. 25 (6), 329–343. 10.1016/0021-9681(72)90027-6 4650929

[B13] Kim E. J. Lee R. K. Lee S. M. Kim D. Y. (2001). General pharmacology of IY-81149, a new proton pump inhibitor. Arzneim. 51 (1), 51–59. 10.1055/s-0031-1300002 11215326

[B14] Kwon D. Chae J. B. Park C. W. Kim Y. S. Lee S. M. Kim E. J. (2001). Effects of IY-81149, a newly developed proton pump inhibitor, on gastric acid secretion *in vitro* and *in vivo* . Arzneim. 51 (3), 204–213. 10.1055/s-0031-1300026 11304936

[B15] Morrish G. A. Pai M. P. Green B. (2011). The effects of obesity on drug pharmacokinetics in humans. Expert Opin. drug metabolism Toxicol. 7 (6), 697–706. 10.1517/17425255.2011.570331 21417960

[B16] Nagase M. Shimada H. Nii M. Ueda S. Higashimori M. Ichikawa K. (2020). Population pharmacokinetic analysis of esomeprazole in Japanese subjects with various CYP2C19 phenotypes. J. Clin. Pharm. Ther. 45 (5), 1030–1038. 10.1111/jcpt.13129 32227647

[B17] Sachs G. Shin J. M. Howden C. W. (2006). Review article: the clinical pharmacology of proton pump inhibitors. Alimentary Pharmacol. Ther. 23 (Suppl. 2), 2–8. 10.1111/j.1365-2036.2006.02943.x 16700898

[B18] Sakurai Y. Hirayama M. Hashimoto M. Tanaka T. Hasegawa S. Irie S. (2007). Population pharmacokinetics and proton pump inhibitory effects of intravenous lansoprazole in healthy Japanese males. Biol. Pharm. Bull. 30 (12), 2238–2243. 10.1248/bpb.30.2238 18057705

[B19] Seo K. A. Lee S. J. Kim K. B. Bae S. K. Liu K. H. Kim D. H. (2012). Ilaprazole, a new proton pump inhibitor, is primarily metabolized to ilaprazole sulfone by CYP3A4 and 3A5. Xenobiotica 42 (3), 278–284. 10.3109/00498254.2011.622416 22022918

[B20] Shen T. Jiang X. Jin Z. Ji Q. Li Q. (2020). The study of intestinal absorption and biodistribution *in vivo* of proton pump inhibitors. Eur. J. Pharm. Biopharm. 149, 135–144. 10.1016/j.ejpb.2020.01.015 32007590

[B21] Shin J. S. Lee J. Y. Cho K. H. Park H. L. Kukulka M. Wu J. T. (2014). The pharmacokinetics, pharmacodynamics and safety of oral doses of ilaprazole 10, 20 and 40 mg and esomeprazole 40 mg in healthy subjects: a randomised, open-label crossover study. Alimentary Pharmacol. Ther. 40 (5), 548–561. 10.1111/apt.12860 25041486

[B22] Tanaka E. (1999). Gender-related differences in pharmacokinetics and their clinical significance. J. Clin. Pharm. Ther. 24 (5), 339–346. 10.1046/j.1365-2710.1999.00246.x 10583696

[B23] Targownik L. E. Fisher D. A. Saini S. D. (2022). AGA clinical practice update on de-prescribing of proton pump inhibitors: expert review. Gastroenterology 162 (4), 1334–1342. 10.1053/j.gastro.2021.12.247 35183361

[B24] Wang H. Lang L. Ou N. Shi R. Hu H. Hu P. (2016). Pharmacokinetics, pharmacodynamics and safety of multiple-infusion ilaprazole in healthy Chinese subjects. Clin. drug Investig. 36 (6), 463–470. 10.1007/s40261-016-0390-2 27067231

[B25] Wang H. Ou N. Lang L. Shi R. Hu P. Jiang J. (2016). Pharmacokinetics and pharmacodynamics of intravenous ilaprazole in healthy subjects after single ascending doses. Xenobiotica 46 (12), 1133–1141. 10.3109/00498254.2016.1156185 26998954

[B26] Wang H. Shao F. Liu X. Xu W. Ou N. Qin X. (2019). Efficacy, safety and pharmacokinetics of ilaprazole infusion in healthy subjects and patients with esomeprazole as positive control. Br. J. Clin. Pharmacol. 85 (11), 2547–2558. 10.1111/bcp.14076 31332820 PMC6848954

[B27] Wang L. Zhou L. Hu H. Lin S. Xia J. (2012). Ilaprazole for the treatment of duodenal ulcer: a randomized, double-blind and controlled phase III trial. Curr. Med. Res. Opin. 28 (1), 101–109. 10.1185/03007995.2011.639353 22070512

