# Supplementary material for: Population pharmacokinetic modeling of ilaprazole in healthy subjects and patients with duodenal ulcer in China
Source: Front Pharmacol. 2024 Jan 10;14:1306222. doi: 10.3389/fphar.2023.1306222 (PMC10805834; doi:10.3389/fphar.2023.1306222)
Supplement: Supplementary file 1 [file Table1.DOCX]

Supplementary Material

# Supplementary Figures


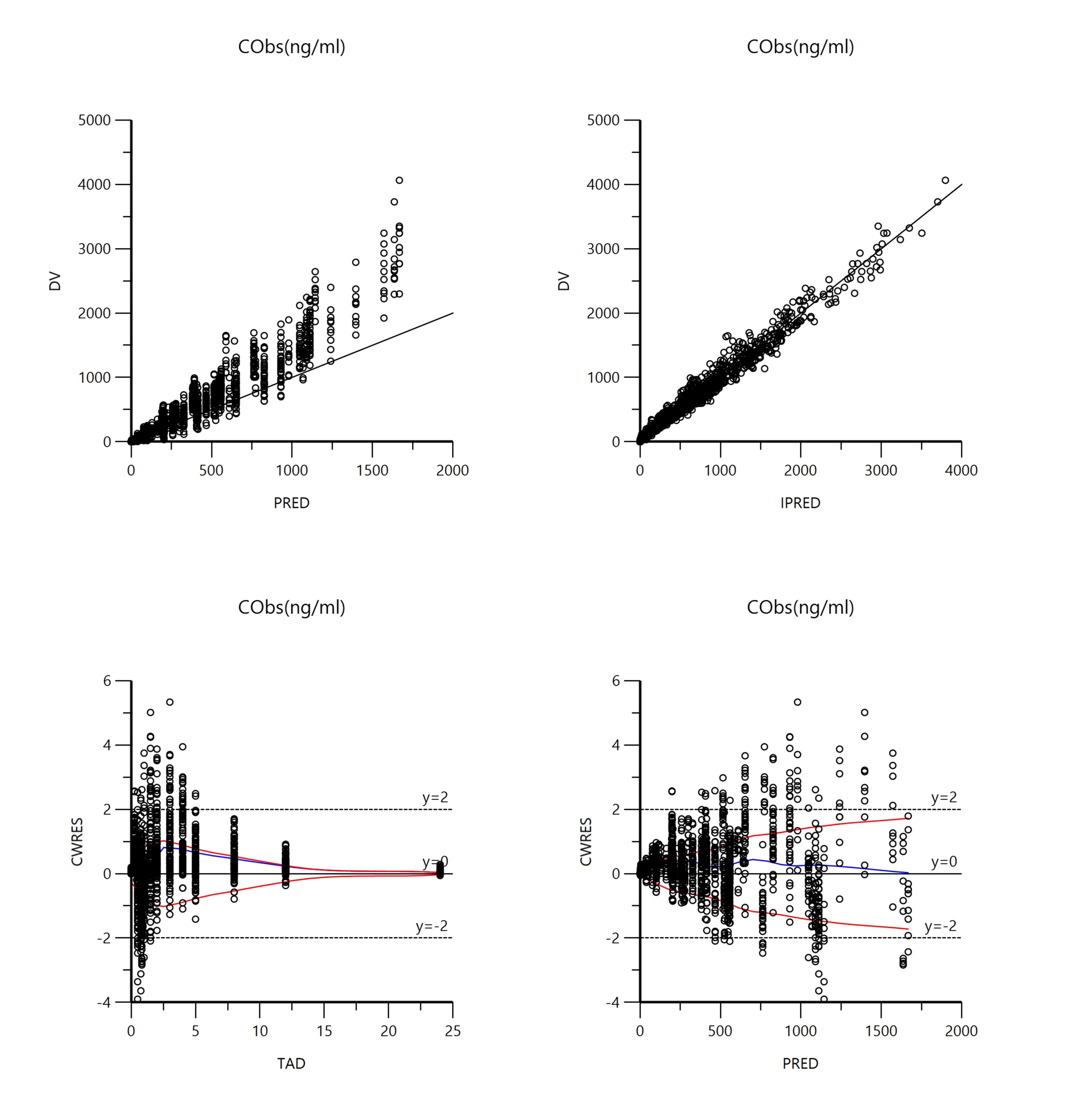


**Figure S1.** Goodness-of-fits plots for the base model with one-compartment: (a) dependent variable versus population prediction plot (DV-PRED) of ilaprazole; (b) dependent variable versus individual prediction plot (DV-IPRED) of ilaprazole; (c) conditional weighted residuals errors versus time after last dose plot (CWRES-TAD) of ilaprazole; (d) conditional weighted residuals errors versus population prediction plot (CWRES-PRED) of ilaprazole.

**
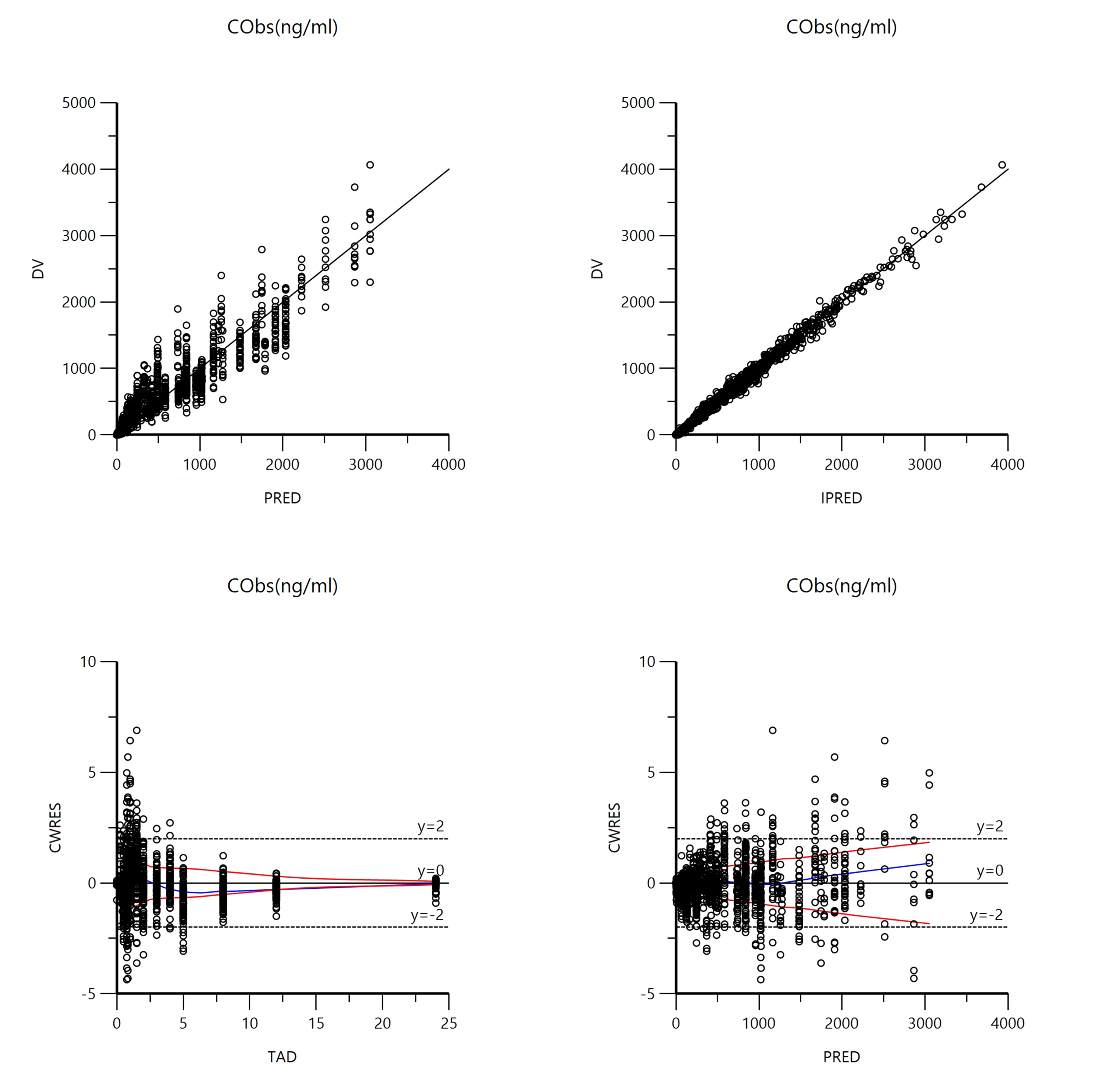
**

**Figure S2.** Goodness-of-fits plots for the base model with two-compartment: (a) dependent variable versus population prediction plot (DV-PRED) of ilaprazole; (b) dependent variable versus individual prediction plot (DV-IPRED) of ilaprazole; (c) conditional weighted residuals errors versus time after last dose plot (CWRES-TAD) of ilaprazole; (d) conditional weighted residuals errors versus population prediction plot (CWRES-PRED) of ilaprazole.


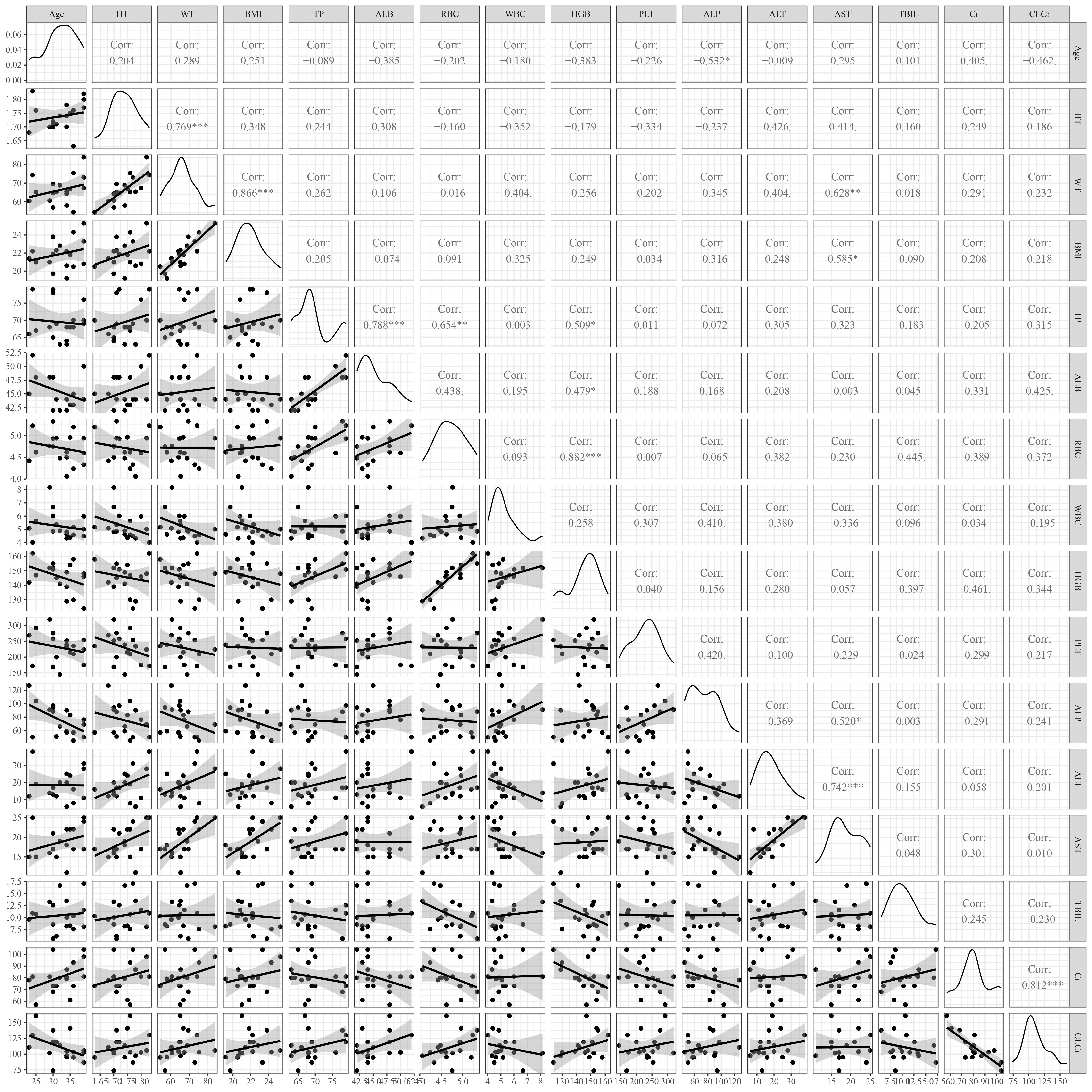


Figure S3. Correlation analysis of continuous covariates.
